# Dynamics of Acute Postsurgical Pain over the Last Decade: A Bibliometric Analysis

**DOI:** 10.1155/2022/8090209

**Published:** 2022-11-07

**Authors:** Zhimin Tan, Yanjie Dong, Qian Li

**Affiliations:** Department of Anesthesiology, West China Hospital, Sichuan University, No. 37 Guo Xue Xiang, Chengdu 610041, Sichuan, China

## Abstract

**Objective:**

Minimizing acute postsurgical pain (APSP) remains a challenge, despite extensive research about it. This study comprehensively analyzed the literature on APSP to assess how the field has developed and where it may go in the future.

**Methods:**

Studies on APSP indexed in the Web of Science Core Collection and published from 2012 to 2021 were assessed for eligibility. Data from included studies were analyzed using CiteSpace, Python, and Microsoft.

**Results:**

Analysis of 5,236 publications on APSP showed that the number of articles per year has increased linearly. The United States leads other countries in terms of the number and centrality of publications. Cocitation analysis suggests that the field focused earlier on the incidence and risk factors of APSP, shifting later to a focus on the reduction and management of adverse outcomes due to APSP. The top-ranked keyword cluster during the study period was “short-term outcomes” (#0), followed by “risk factors” (#1). The strongest burst occurred for the keyword “combination,” followed by “multimodal analgesia.” The most recent burst occurred for the keywords “regional analgesia,” “opioid use,” “erector spinae plane block,” and “infiltration.”

**Conclusions:**

Hotspots in APSP research since 2012 have been incidence, risk factors, and control of negative outcomes. Future research is likely to concentrate on the use of opioids and technological innovations in regional anesthesia. Our findings may help APSP researchers and clinicians understand their field, optimize clinical practice, and plan future research.

## 1. Introduction

Approximately 312 million surgeries are performed around the world each year [1], and acute postsurgical pain (APSP) after these procedures remains a major challenge, despite more than 30 years of research about it [2]. One study suggests that more than 80% of surgical patients experience APSP, which is moderate or severe in 75% of them [3]. APSP increases the risk of poor emotional state, respiratory disease, cardiovascular complications, and systemic stress responses [4, 5]. The longer the duration of severe APSP, the greater the risk of chronic postsurgical pain [6, 7]. In this way, APSP reduces the quality of life, which became evident in the recent opioid crisis [8, 9].

In 2012, the American Society of Anesthesiologists updated its guidelines for managing APSP, and it began to recommend multimodal analgesia [10]. In the ensuing decade, multimodal analgesia as well as the approach of “enhanced recovery after surgery” were widely implemented in clinics. During this period, opioid-free anesthesia and ultrasound-guided nerve blocks were introduced [11–13]. These advances and further research helped to reduce APSP incidence by more than 30% since 2014 [3, 14]. Nevertheless, APSP remains common, highlighting the need to assess how the field of APSP has evolved in order to understand where it can be directed in the future.

An effective way to assess the evolution of a research field is through analysis of the countries, institutions, journals, and authors who have published in the field, together with the keywords describing those publications [15–17]. Such bibliometric analysis can summarize development trends and stimulate innovation and creativity [18].

Based on the Web of Sciences, the literature on APSP grew by more than 11,000 since 1992, when the first acute pain management guidelines were published, and by more than 6,000 in the last decade alone. Given such voluminous literature, a bibliometric analysis may provide more insights than a traditional literature review. Therefore, the present work performed a bibliometric analysis of APSP research to examine its development over the last several decades and predict its future.

## 2. Materials and Methods

### 2.1. Literature Search

A comprehensive search of the Web of Science Core Collection database was conducted on 10 February 2022 using the search string TS = (“acute postoperative pain”) AND (“acute postsurgical pain”). The publication window for eligible studies was defined as January 1, 2012, to December 31, 2021. The initial date was chosen based on the publication of the APSP guidelines by the American Society of Anesthesiologists and because the last decade has seen the largest annual increase in APSP publications since 1992. Eligible studies during this publication period had to be research articles or review articles published in English and indexed in the Web of Science Core Collection. Articles that did not meet these criteria or that were published as “corrections,” “editorial material,” “letters,” “meeting abstracts,” “proceedings papers,” or “book chapters” were excluded.

### 2.2. Data Extraction and Bibliometric Analysis

The following data were extracted from each article: authors, title, abstract, institution, country, keywords, and references. Bibliometric data were visually analyzed using CiteSpace, Python, and Microsoft. CiteSpace software creates a comprehensive network based on a time series of annual publications to model the structure of knowledge [19]. The size of the nodes in the network reflects the frequency of co-occurrence [20], and connections between nodes indicate co-occurrence relationships. In a time-slice sequence, each node is represented by a series of citation tree rings. The purple outer ring suggests a high degree of centrality (> 0.1), a measure associated with the conversion potential of scientific contributions [21]. Our analysis included burst detection in order to detect burst keywords. Annual and total numbers of publications and citations were analyzed using Microsoft Excel. Growth in the number of publications was modeled using the linear equation *f*(*X*) = *aX* + *b*, where *X* referred to the year of publication and *f*(*X*) to the number of studies published in that year. Python was applied to analyze geographically where APSP research was performed and where research collaborations formed.

## 3. Results

**Figure 1:**
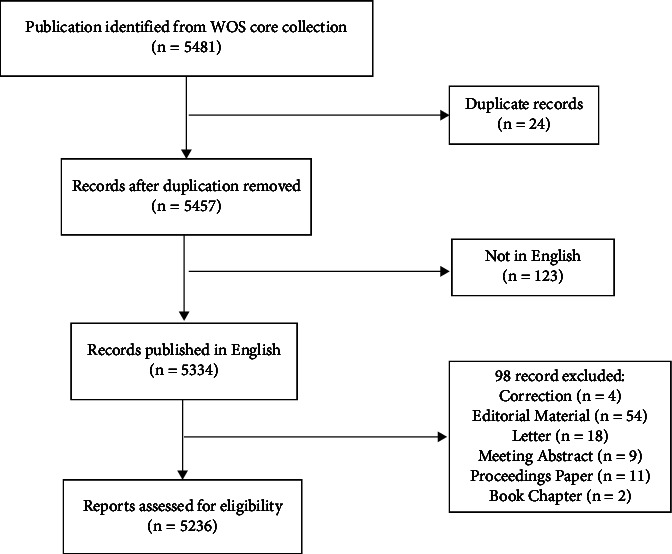
Flow chart of study inclusion.

Initially, 5,481 potentially eligible studies were found, of which 5,236 were retained after rigorous screening (Figure 1). Relevant data were downloaded from all studies on 10 February 2022 to ensure data uniformity and accuracy. Details and reasons for exclusion studies are provided in Supplementary Tables 1–3.

**Figure 2:**
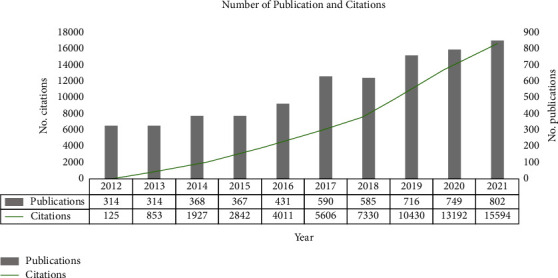
Absolute numbers of publications related to acute postsurgical pain and numbers of citations to those publications, 2012–2021. Notes: the data has been taken from the publication year to the retrieved date (February 10, 2022).

**Figure 3:**
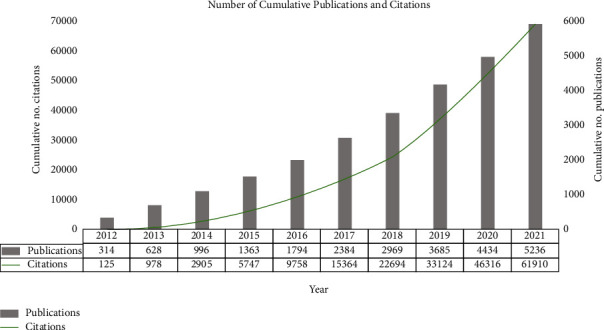
Cumulative numbers of publications related to acute postsurgical pain and numbers of citations to those publications, 2012–2021. Notes: the data has been taken from the publication year to the retrieved date (February 10, 2022).

**Figure 4:**
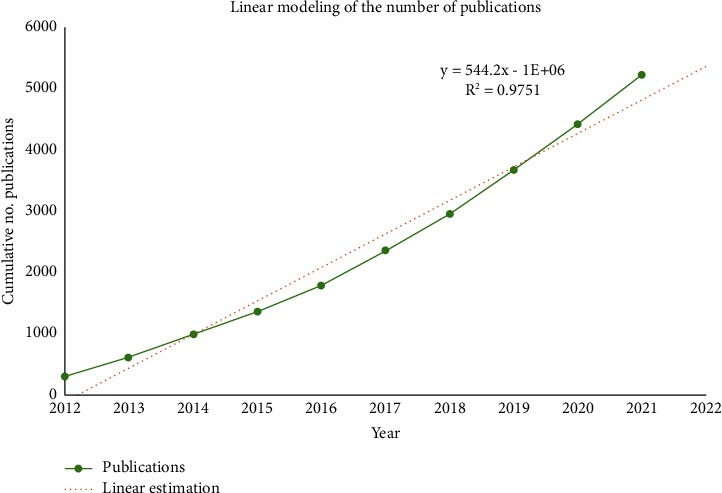
Linear modeling of the cumulative number of publications related to acute postsurgical pain, 2012–2021.

**Figure 5:**
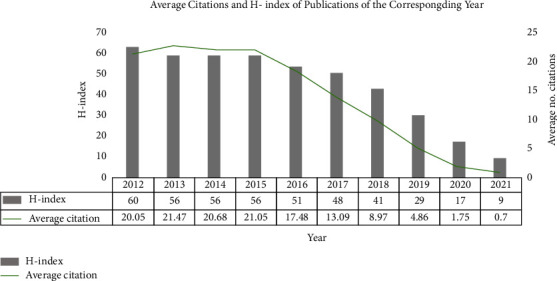
Average citations to publications about acute postsurgical pain and H-index of such publications by year, 2012–2021. Notes: the data has been taken from the publication year to the retrieved date (February 10, 2022); H-index: the H-index for the corresponding year was defined as all publications in the APSP field during this period in which at least H papers were cited at least H times.

The Number of APSP publications and citations of them has increased steadily over the past decade (Figures 2 and 3). Modeling showed a significant, positive, linear correlation between the number of papers and year (*R*^2^ = 0.9751, Figure 4). The year 2021 accounted for the largest proportion of APSP publications of all the years in the study period (802/5236, 15.3%), with 2.55 times more than in 2012 (314, 5.9%). By February 10, 2022, the APSP publications in our dataset had been cited 61,910 times, with 12.04 citations per article. The overall H-index for APSP publications during the study period was 87, this index peaked at 60 in 2012 (Figure 5).

**Figure 6:**
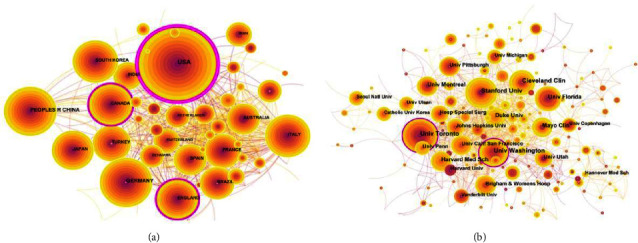
Maps of collaborative research on acute postsurgical pain among (a) countries and (b) institutions, 2012–2021.

**Figure 7:**
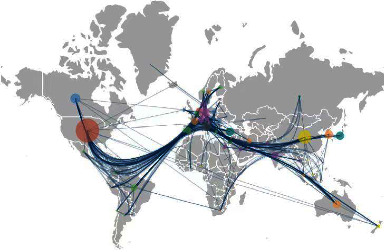
Cooperation network map of APSP.

Approximately 400 institutions from 122 countries published APSP research during the study period. The research concentrated on a few regions of the world, namely western Europe, North America, and Asia, and particularly in a few countries, namely the USA, China, Germany, and Canada (Figures 6 and 7, Table 1). Centricity was substantially higher for the USA, Canada, and the UK than for the other top-publishing countries (Table 1), suggesting that research in these countries is more likely to function as a bridge toward “turning points” in the APSP field [22]. Indeed, all 10 top-publishing institutions were in the USA and Canada, accounting for 8% of the global total (441/5236). The University of Toronto published the most APSP publications during the study period, but the University of Washington ranked first in centrality.

**Figure 8:**
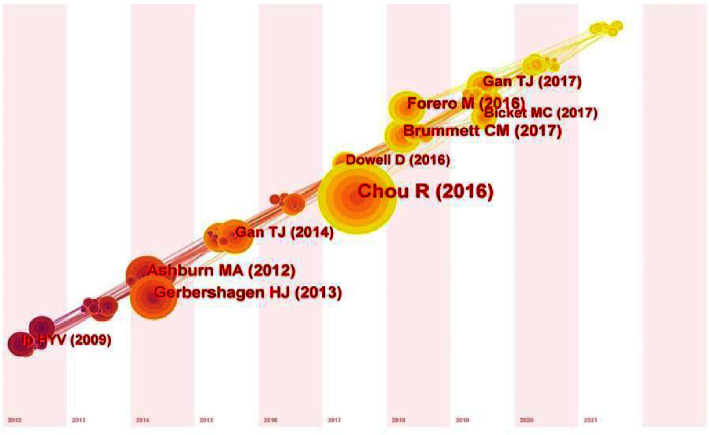
Time-zone depiction of cocited publications on acute postsurgical pain, 2012–2021.

Next, we examined the cocitations of APSP in 2012–2021 to identify research that has been particularly influential in the development of this field. The top 10 cocited publications during the study period were three guidelines, four reviews, and three clinical studies. The top ranking of cocitations was the clinical practice guidelines updated by the American Pain Society in 2016, with a total of 174 co-citations (Table 2) [23]. Time-zone analysis of the cocitation network (Figure 8) showed that earlier APSP studies focused on the prediction of risk factors for postoperative pain [22, 29]. Subsequently, the research focus shifted to the management of postoperative pain [24, 25, 27].

**Figure 9:**
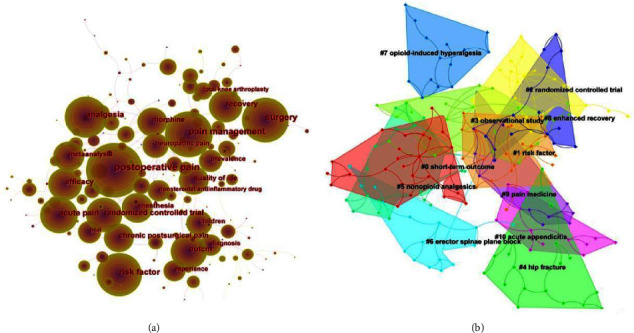
The (a) co-occurrence and (b) clustering of keywords in publications on acute postsurgical pain, 2012–2021.

Hotspots of APSP research emerged from analysis of the 172 keywords that appeared more than three times among APSP publications during the study period (Figure 9(a)). To reduce biases in our fully automated bibliometric analysis, we manually merged the results for the following pairs of closely related keywords: “risk” and “risk factors,” “pain management” and “management,” as well as “postsurgical pain” and “perioperative pain.” The most frequent keyword was “postoperative pain” (932 times), followed by “management” (706) and “surgery” (589). Also frequent were keywords related to perioperative pain management, including “analgesia” (370), “risk factor” (307), and “efficacy” (250). The most frequent clusters of keywords were “short-term outcomes” (#0), “risk factors” (#1), and “randomized controlled trials” (#2) (Figure 9(b)).

**Figure 10:**
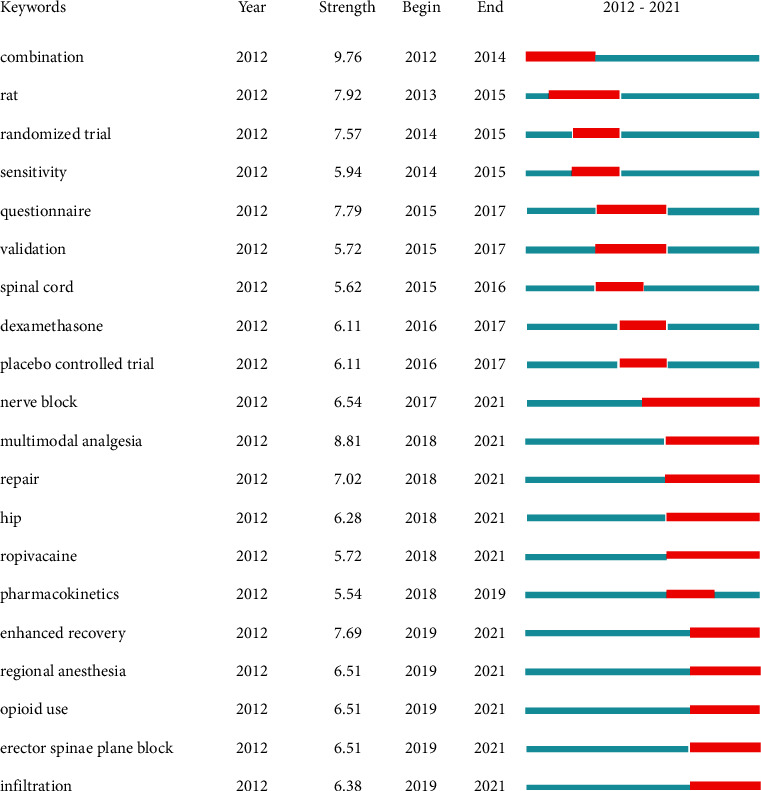
The 20 keywords from publications on acute postsurgical pain show the strongest bursts of citations. Notes: the lengths of red bursts on the blue timelines are proportional to their duration.

Finally, we analyzed bursts in keywords at different times in the study period in order to track the evolving focus of APSP research (Figure 10). The keyword with the strongest burst was “combination” (9.76), followed by “multimodal analgesia” (8.81) and “rat” (7.92). From 2013–2017, bursts occurred for keywords related mainly to study methods, such as “randomized follow-up,” “questionnaire,” and “validation.” After 2018, bursts occurred for keywords related to frontier hotspots of research, including “regional analgesia,” “opioids use,” “erector spinae plane block,” and “infiltration.” This phenomenon suggests an increasing emphasis on clinical applications and research of practical value.

## 4. Discussion

### 4.1. General Trends in APSP Research

Our analysis of APSP research published between 2012 and 2021 and indexed in the Web of Science Core Collection suggests that the volume of research has increased linearly during the period, indicating a field that likely will continue to grow. On the other hand, our analysis of the H-index and citation frequency of those publications increased until 2015, then decreased. While it can take several years for a publication's impact to be reflected in citations and the H-index [30, 31], our analysis suggests that future ASPS research should aim to follow best practices and pursue high-quality evidence.

We found that the USA has occupied the leading position in APSP research, in terms of both volume and centrality of publications, with support from a collaborative network involving Australia, the UK, Canada, and France. While countries in Europe and North America collaborate closely in this field, countries in Asia appear to collaborate only loosely. These geographical differences in APSP research highlight the need to ensure that studies in this field are conducted in a way that captures real-world regional differences in healthcare systems and clinic demographics of study populations. In this sense, collaborations across institutions and national borders are extremely important.

### 4.2. Evolution of APSP Research

Analysis of the APSP publications that were highly cocited during the study period allowed us to trace the trajectory of research in this area. Early highly cited literature revealed the high incidence and serious adverse outcomes of APSP. For example, a survey in 2014 suggested that more than 80% of surgical patients experienced APSP [3] higher than the 57–59% reported 20 (in 2003) and 30 (in 1995) years ago [32, 33]. Poor control of APSP is strongly associated with impaired function, delayed recovery, and prolonged opioid use [26].

This led to a later focus on how to prevent APSP and improve prognoses, such as through timely identification of risk factors and targeted analgesic interventions. A systematic review of studies involving more than 23,000 patients identified preoperative pain, anxiety, age, and type of surgery as the four most important risk factors for APSP [29]. A cohort study of more than 115,000 patients highlighted the need to tailor pain management to the surgical procedure in order to avoid over- or under-analgesia [22].

Partly as a result of these studies, substantial research has examined how to optimize perioperative pain management and the use of opioids. For example, 2016 saw the first report of erector spinae plane block in patients undergoing video-assisted thoracoscopic wedge resection of the right upper lobe [25]. During general anesthesia, the patient was injected with only 250 *μ*g fentanyl and 1 mg hydromorphone for additional analgesia. Postoperatively, the patient reported a pain score of 0, and no further opioid therapy was required. This and related studies have shown that targeted nerve blocks can adequately control thoracic neuropathic pain, which is insensitive to both oral and intravenous drug therapy, and it can reduce or eliminate the need for perioperative opioids.

Nevertheless, the increasing popularity of multimodal analgesia and innovations with local analgesics have led researchers to pay increasing attention to opioid management. Analysis of a cohort of more than 36,000 surgical patients showed that 6% continued to use opioids 90 days after surgery [24]. This demonstrates that persistent postoperative opioid use is not uncommon, and it may be consistently underestimated in the field. That analysis highlighted that factors other than postoperative pain were also associated with persistent use of opioids, such as preoperative smoking, alcohol, substance abuse, and emotional disturbances. Future research should explore whether targeting these risk factors may reduce the long-term use of opioids.

### 4.3. Future Trends in APSP Research

Analysis of keyword bursts allowed us to identify research areas that have contributed significantly to reducing the incidence of APSP in the past decade, as well as areas poised to become important in the future of the field. The keyword “combination” showed the strongest burst, which lasted from 2012 to 2014. The second-ranked “multimodal analgesia” showed a burst from 2018 to 2021. Bursts after 2017 involved keywords related mostly to local analgesia, including “nerve block,” “regional anesthesia,” “erector spinae plane block,” and “infiltration.” By 2019, “opioid use” experienced a burst, reflecting growing concern about the opioid crisis.

These observations suggest that the exploration of multimodal analgesia has been the mainstay of APSP research and that local analgesia and opioid management are future directions of the field. The introduction of multimodal analgesia and the availability of perioperative ultrasonography has led to substantial progress in regional analgesia. For example, the erector spinae plane block, first reported in 2016, has since been applied to an increasingly broad range of clinical situations, including complex regional pain syndromes and herpes zoster [34–36]. Future research on multimodal analgesia seems likely to bring additional regional anesthesia techniques. On the other hand, a multimodal analgesia is also an effective tool for the implementation of the perioperative opioid-saving strategy. The surge of the keyword “opioid use” in 2019 may be related to the growing concerns about the opioid crisis. In 2017, nearly 10 times more people died in the USA from fentanyl and other synthetic opioids than in 2010 [37]. At the same time, research continued to identify and optimize alternatives to opioids as analgesics. While several nonopioid analgesics effectively reduce postoperative pain [38], dexmedetomidine has been associated with bradycardia and hypotension; nonsteroidal anti-inflammatory drugs, with nephrotoxicity; and certain combinations of nonopioids, with respiratory depression and cognitive dysfunction [39–41]. There is no widely accepted “gold standard” for the implementation of opioid-free anesthesia, the effectiveness and safety of this strategy still await multilevel validation. Thus, tremendous potential exists for the application and management of opioids as pain-related research abounds [42, 43].

## 5. Limitations

Our literature search was limited to the Web of Science because several of the scientometric tools that we applied, particularly CiteSpace, are not currently compatible with PubMed or the Cochrane Library. Nevertheless, journals indexed in the Web of Science are recognized as being of good quality. Our analysis may underestimate citation frequency and H-index for the last few years of the study period because of the lag between a publication's appearance and when it begins to be cited. For the same reason, our keyword analysis may be less accurate toward the end of the study period. Our data extraction and analysis were entirely automated, in contrast to the manual processes in a traditional meta-analysis or systematic review. This may create biases in our analyses, which we tried to minimize by manually merging the results for closely related keywords. Future studies should aim to draw on a wider range of literature databases and perform more sophisticated analyses that can subtly differentiate research subareas.

## 6. Conclusions

This study has traced the development of the literature, explored hot areas, and predicted future frontiers in the APSP field. Research in this area is rapidly evolving. Researchers earlier focused on the incidence of ASPS and its risk factors. As more risk factors were identified, researchers shifted to focus on controlling and managing the negative effects of APSP. Local analgesic techniques, an important component of multimodal analgesia, are rapidly becoming a frontier of research. At the same time, the application of multimodal analgesia is driving research in opioid management. Our bibliometric analysis provides useful insights into the past and future of APSP research. It also illustrates the potential advantages of scientometrics over traditional literature reviews for understanding research fields.

## Figures and Tables

**Table 1:** Countries and institutions publishing the most articles about acute postsurgical pain, 2012–2021.

Rank	Country	No. publications	Centricity	Institution	No. publications	Centricity
1	USA	1,236	0.18	University of Toronto (Canada)	63	0.14
2	China	435	0.04	Stanford University (USA)	57	0.08
3	Germany	266	0.04	University of Washington (USA)	50	0.18
4	Canada	202	0.15	Cleveland Clinic (USA)	48	0.08
5	South Korea	201	0.01	Duke University (USA)	42	0.03
6	Italy	190	0.05	Harvard Medical School (USA)	40	0.03
7	Japan	187	0	University of Florida (USA)	38	0.01
8	Turkey	178	0	Mayo Clinic (USA)	36	0.03
9	United Kingdom	168	0.13	University of Montreal (Canada)	35	0.03
10	France	122	0.04	The University of California at San Francisco (USA)	32	0.05

*Note.* The data has been taken from the publication year to the retrieved date (February 10, 2022).

**Table 2:** Top 10 cocited references related to acute postsurgical pain, 2012–2021.

Rank	Cocitation counts	Centricity	Author	Year	Title	Journal
1	174	0.44	Chou	2016	Management of Postoperative Pain: A Clinical Practice Guideline from the American Pain Society, the American Society of Regional Anesthesia and Pain Medicine, and the American Society of Anesthesiologists' Committee on Regional Anesthesia, Executive Committee, and Administrative Council [23]	The Journal of Pain
2	56	0.28	Ashburn	2012	Practice Guidelines for Acute Pain Management in the Perioperative Setting: An Updated Report by the American Society of Anesthesiologists Task Force on Acute Pain Management [10]	Anesthesiology
3	54	0.21	Gerbershagen	2013	Pain Intensity on the First Day after Surgery: A Prospective Cohort Study Comparing 179 Surgical Procedures [22]	Anesthesiology
4	51	0.12	Brummett	2017	New Persistent Opioid Use after Minor and Major Surgical Procedures in US adults [24]	Journal of the American Medical Association Surgery
5	49	0.13	Forero	2016	The Erector Spinae Plane Block: A Novel Analgesic Technique in Thoracic Neuropathic Pain [25]	Regional Anesthesia and Pain Medicine
6	42	0.5	Gan	2017	Poorly Controlled Postoperative Pain: Prevalence, Consequences, and Prevention [26]	Journal of Pain Research
7	41	0.03	Gan	2014	Incidence, Patient Satisfaction, and Perceptions of Postsurgical Pain: Results from a US National Survey [3]	Current Medical Research and Opinion
8	29	0.26	Bicket	2017	Prescription Opioid Analgesics Commonly Unused after Surgery: A Systematic Review [27]	Journal of the American Medical Association Surgery
9	29	0.22	Dowell	2016	CDC Guideline for Prescribing Opioids for Chronic Pain—United States, 2016 [28]	MMWR. Recommendations and Reports
10	29	0.22	Ip	2009	Predictors of Postoperative Pain and Analgesic Consumption: A Qualitative Systematic Review [29]	Anesthesiology

*Note.* The data has been taken from the publication year to the retrieved date (February 10, 2022).

## Supplementary Materials

Supplementary Materials
*Supplementary Table 1.* Details of excluded duplicates. *Supplementary Table 2.* Details of excluded literature in non-English languages. *Supplementary Table 3*. Details of literature excluded due to type of literature.
