# Supplementary material for: Dynamics of Acute Postsurgical Pain over the Last Decade: A Bibliometric Analysis
Source: Pain Res Manag. 2022 Nov 7;2022:8090209. doi: 10.1155/2022/8090209 (PMC9663218; doi:10.1155/2022/8090209)
Supplement: Supplementary Materials — Supplementary Table 1. Details of excluded duplicates. Supplementary Table 2. Details of excluded literature in non-English languages. Supplementary Table 3. Details of literature excluded due to type of literature. [file 8090209.f1.docx]

**Supplementary table 1 Details of excluded duplicates**

| **Rank** | **Article Title** | **Language** | **Document Type** | **DOI** |
| --- | --- | --- | --- | --- |
| 1 | Acute and Chronic Postsurgical Pain After Living Liver Donation: Incidence and Predictors | English | Article | 10.1002/lt.23949 |
| 2 | Acute and chronic postsurgical pain after living liver donation: Incidence and predictors | English | Letter | 10.1002/lt.24125 |
| 3 | Agreement of the Professional Association of German Anesthesiologists and the Professional Association of German Surgeons for the Organization of Postoperative Pain Therapy for Surgical Patients (revised version 2019) | German | Article | 10.1007/s00113-019-0701-1 |
| 4 | Agreement of the Professional Association of German Anesthesiologists and the Professional Association of German Surgeons for the Organization of Postoperative Pain Therapy for Surgical Patients (revised version 2019) | German | Article | 10.1007/s00482-019-0394-y |
| 5 | Agreement of the Professional Association of German Anesthesiologists and the Professional Association of German Surgeons for the Organization of Postoperative Pain Therapy for Surgical Patients (revised version 2019) | German | Article | 10.1007/s00104-019-0995-0 |
| 6 | Agreement of the Professional Association of German Anesthesiologists and the Professional Association of German Surgeons for the Organization of Postoperative Pain Therapy for Surgical Patients (revised version 2019) | German | Article | 10.1007/s00101-019-0629-4 |
| 7 | Caffeine as an analgesic adjuvant for acute pain in adults | English | Review | 10.1002/14651858.CD009281.pub3 |
| 8 | Caffeine as an analgesic adjuvant for acute pain in adults | English | Review | 10.1002/14651858.CD009281.pub2 |
| 9 | Does Gabapentin make any difference in Post-Operative Pain in Modified Radical Mastectomy patients? | English | Article |  |
| 10 | Does Gabapentin make any difference in Post-Operative Pain in Modified Radical Mastectomy patients? | English | Article |  |
| 11 | EFFICACY AND SAFETY OF IBUPROFEN PLUS PARACETAMOL IN A FIXED-DOSE COMBINATION FOR ACUTE POSTOPERATIVE PAIN IN ADULTS: META-ANALYSIS AND A TRIAL SEQUENTIAL ANALYSIS | English | Meeting Abstract |  |
| 12 | Efficacy and Safety of Ibuprofen Plus Paracetamol in a Fixed-Dose Combination for Acute Postoperative Pain in Adults: Meta-Analysis and a Trial Sequential Analysis | English | Article | 10.1007/s40263-020-00777-7 |
| 13 | Italian intersociety consensus on prevention, diagnosis, and treatment of delirium in hospitalized older persons | English | Article | 10.1007/s11739-017-1705-x |
| 14 | Italian intersociety consensus on prevention, diagnosis, and treatment of delirium in hospitalized older persons | English | Article | 10.1016/j.eurger.2017.06.010 |
| 15 | Non-pharmacological interventions for preventing delirium in hospitalised non-ICU patients | English | Review | 10.1002/14651858.CD013307.pub2 |
| 16 | Non-pharmacological interventions for preventing delirium in hospitalised non-ICU patients | English | Review | 10.1002/14651858.CD013307.pub3 |
| 17 | Pain inhibition by opioids-new concepts | German | Article | 10.1007/s00101-018-0528-0 |
| 18 | Pain inhibition by opioids-new concepts | German | Article | 10.1007/s00482-019-0386-y |
| 19 | Perioperative analgesia with nonopioid analgesics Joint interdisciplinary consensus-based recommendations of the German Pain Society, the German Society of Anaesthesiology and Intensive Care Medicine and the German Society of Surgery | German | Article | 10.1007/s00104-021-01421-w |
| 20 | Perioperative analgesia with nonopioid analgesics Joint interdisciplinary consensus-based recommendations of the German Pain Society, the German Society of Anaesthesiology and Intensive Care Medicine and the German Society of Surgery | German | Article | 10.1007/s00101-021-01010-w |
| 21 | Perioperative intravenous ketamine for acute postoperative pain in adults* | English | Editorial Material |  |
| 22 | Perioperative intravenous ketamine for acute postoperative pain in adults | English | Review | 10.1002/14651858.CD012033.pub4 |
| 23 | Postoperative pain management | English | Review | 10.12968/hmed.2015.76.10.570 |
| 24 | Postoperative pain management | German | Review |  |
| 25 | Prevention of Venous Thromboembolism in Gynecologic Surgery ACOG Practice Bulletin, Number 232 | English | Editorial Material | 10.1097/AOG.0000000000004446 |
| 26 | Prevention of Venous Thromboembolism in Gynecologic Surgery ACOG Practice Bulletin, Number 232 | English | Article | 10.1097/AOG.0000000000004445 |
| 27 | Psychological interventions for acute pain after open heart surgery | English | Review | 10.1002/14651858.CD009984.pub2 |
| 28 | Psychological interventions for acute pain after open heart surgery | English | Review | 10.1002/14651858.CD009984.pub3 |
| 29 | Research design considerations for chronic pain prevention clinical trials: IMMPACT recommendations | English | Review | 10.1097/j.pain.0000000000000191 |
| 30 | Research design considerations for chronic pain prevention clinical trials: IMMPACT recommendations | English | Review | 10.1097/PR9.0000000000000895 |
| 31 | Single dose oral celecoxib for acute postoperative pain in adults | English | Review | 10.1002/14651858.CD004233.pub4 |
| 32 | Single dose oral celecoxib for acute postoperative pain in adults | English | Review | 10.1002/14651858.CD004233.pub3 |
| 33 | Single dose oral etoricoxib for acute postoperative pain in adults | English | Review | 10.1002/14651858.CD004309.pub3 |
| 34 | Single dose oral etoricoxib for acute postoperative pain in adults | English | Review | 10.1002/14651858.CD004309.pub4 |
| 35 | Single dose oral ibuprofen plus codeine for acute postoperative pain in adults | English | Review | 10.1002/14651858.CD010107.pub3 |
| 36 | Single dose oral ibuprofen plus codeine for acute postoperative pain in adults | English | Review | 10.1002/14651858.CD010107.pub2 |
| 37 | Systemic antibiotics for symptomatic apical periodontitis and acute apical abscess in adults | English | Review | 10.1002/14651858.CD010136.pub2 |
| 38 | Systemic antibiotics for symptomatic apical periodontitis and acute apical abscess in adults | English | Review | 10.1002/14651858.CD010136.pub3 |
| 39 | The ACTTION-APS-AAPM Pain Taxonomy (AAAPT) Multidimensional Approach to Classifying Acute Pain Conditions | English | Review | 10.1093/pm/pnx019 |
| 40 | The ACTTION-APS-AAPM Pain Taxonomy (AAAPT) Multidimensional Approach to Classifying Acute Pain Conditions | English | Article | 10.1016/j.jpain.2017.02.421 |
| 41 | Treatment and prevention of pouchitis after ileal pouch-anal anastomosis for chronic ulcerative colitis | English | Review | 10.1002/14651858.CD001176.pub4 |
| 42 | Treatment and prevention of pouchitis after ileal pouch-anal anastomosis for chronic ulcerative colitis | English | Review | 10.1002/14651858.CD001176.pub3 |
| 43 | Treatment and prevention of pouchitis after ileal pouch-anal anastomosis for chronic ulcerative colitis | English | Review | 10.1002/14651858.CD001176.pub5 |

**Supplementary table 2 Details of excluded literature in non-English languages**

| **Rank** | **Article Title** | **Language** | **Document Type** | **DOI** |
| --- | --- | --- | --- | --- |
| 1 | Pelvic neuropathic pain (differential diagnosis) | Czech | Article | 10.48095/cccg2021279 |
| 2 | Chronic pain after cesarean: Impact and risk factors associated | French | Article | 10.1016/j.annfar.2013.08.007 |
| 3 | Intraoperative pain monitoring: Update and future directions | French | Article | 10.1016/j.anrea.2018.03.002 |
| 4 | Perioperative management of breast cancer surgery | French | Article | 10.1016/j.anrea.2021.02.006 |
| 5 | Ultrasound and computed tomography in retrocecal appendicitis | French | Article | 10.1016/j.frad.2014.04.002 |
| 6 | Chronic pain after surgery: State of the art | French | Article | 10.1016/j.lpm.2014.09.025 |
| 7 | Acute aortic syndromes | French | Article | 10.1016/j.lpm.2018.02.001 |
| 8 | Analgesia for knee surgery | French | Review | 10.1016/j.anrea.2019.10.004 |
| 9 | Perioperative stroke | French | Review | 10.1016/j.anrea.2020.04.005 |
| 10 | Perioperative management for total hip arthroplasty | French | Review | 10.1016/j.anrea.2021.04.002 |
| 11 | Postoperative complications after major lung resection | French | Review | 10.1016/j.rmr.2018.09.004 |
| 12 | Sigmoid volvulus in the aged Representative case of a rare constellation of unclear abdomen | German | Article | 10.1007/s00053-019-0354-0 |
| 13 | Anatomic reconstruction of the anterior cruciate ligament in single bundle technique | German | Article | 10.1007/s00064-012-0227-y |
| 14 | Correction of posttraumatic lower leg deformities using the Taylor Spatial Frame | German | Article | 10.1007/s00064-013-0233-8 |
| 15 | Surgical treatment of sternoclavicular joint instability with tenodesis | German | Article | 10.1007/s00064-014-0310-7 |
| 16 | Treatment concept for a traumatic lesion of the prepatellar bursa | German | Article | 10.1007/s00064-015-0414-8 |
| 17 | Total ankle arthroplasty with simultaneous subtalar fusion | German | Article | 10.1007/s00064-017-0498-4 |
| 18 | Wide awake hand surgery based on application examples | German | Article | 10.1007/s00064-018-0544-x |
| 19 | Patellar tendon ruptures. Internal bracing and augmentation technique | German | Article | 10.1007/s00064-018-0585-1 |
| 20 | Removal of epidural catheter under dual antiplatelet therapy following acute coronary syndrome | German | Article | 10.1007/s00101-012-2067-4 |
| 21 | Regional anesthesia in patients with pre-existing infections or immunosuppression | German | Article | 10.1007/s00101-012-2097-y |
| 22 | Pre-existing pain as comorbidity in postoperative acute pain service | German | Article | 10.1007/s00101-013-2224-4 |
| 23 | Update on preemptive analgesia. Options and limits of preoperative pain therapy | German | Article | 10.1007/s00101-013-2225-3 |
| 24 | Rare complication after endoscopic discectomy | German | Article | 10.1007/s00101-013-2279-2 |
| 25 | Management of patients with chronic pain in acute and perioperative medicine An interdisciplinary challenge | German | Article | 10.1007/s00101-019-00708-2 |
| 26 | Perioperative measures for prevention of phantom pain: an evidence-based approach to risk reduction | German | Article | 10.1007/s00101-020-00810-w |
| 27 | Safety and monitoring of patient-controlled intravenous analgesia Clinical practice in German hospitals | German | Article | 10.1007/s00101-020-00907-2 |
| 28 | Cannabis and cannabinoids for the treatment of acute and chronic pain | German | Article | 10.1007/s00101-021-00994-9 |
| 29 | Operation time for suprapubic transumbilical cholecystectomy. Results of a prospective randomized trial | German | Article | 10.1007/s00104-014-2958-9 |
| 30 | Peritoneal adhesion formation | German | Article | 10.1007/s00104-014-2975-8 |
| 31 | Individualized or standard approach to the abdomen. Currently available data | German | Article | 10.1007/s00104-016-0221-2 |
| 32 | Conservative and surgical ileus treatment | German | Article | 10.1007/s00104-017-0438-8 |
| 33 | Antibiotic treatment vs. appendectomy for non-perforated appendicitis in adults | German | Article | 10.1007/s00104-018-0756-5 |
| 34 | Appendectomy: open versus laparoscopic versus single port. Evidence for choice of surgical procedure | German | Article | 10.1007/s00104-018-0758-3 |
| 35 | Current aspects of pain management during and after dermatologic surgery | German | Article | 10.1007/s00105-019-04486-1 |
| 36 | Pain therapy options in trauma and emergency surgery | German | Article | 10.1007/s00113-013-2516-9 |
| 37 | Osteosynthesis of distal fibular fractures with IlluminOss | German | Article | 10.1007/s00113-016-0285-y |
| 38 | Vertebral stability in management of spinal metastases. Criteria and strategies for operative interventions | German | Article | 10.1007/s00132-012-1908-9 |
| 39 | Acromioclavicular injuries in professional athletes | German | Article | 10.1007/s00132-013-2148-3 |
| 40 | Perioperative pain management: what is evidence based? | German | Article | 10.1007/s00132-014-3039-y |
| 41 | Structured rehabilitation after lumbar spine surgery. Subacute treatment phase | German | Article | 10.1007/s00132-014-3051-2 |
| 42 | Postoperative pain assessment and management. Current aspects with special emphasis on cardiac surgery | German | Article | 10.1007/s00398-017-0183-9 |
| 43 | External validity of pain-linked functional interference. Are we measuring what we want to measure? | German | Article | 10.1007/s00482-012-1154-4 |
| 44 | Quality management in acute pain therapy. Results from a survey of certified hospitals | German | Article | 10.1007/s00482-012-1205-x |
| 45 | Oral therapy algorithm for the treatment of postoperative pain. A prospective observational study | German | Article | 10.1007/s00482-012-1279-5 |
| 46 | Transition from acute to chronic postsurgical pain. Physiology, risk factors and prevention | German | Article | 10.1007/s00482-012-1287-5 |
| 47 | Perioperative pain management for abdominal and thoracic surgery | German | Article | 10.1007/s00482-014-1420-8 |
| 48 | Psychological prophylaxis training for coping with postoperative pain. Long-term effects | German | Article | 10.1007/s00482-014-1476-5 |
| 49 | Piritramide versus oxycodone for patient-controlled intravenous analgesia. Opioid-induced side effects | German | Article | 10.1007/s00482-014-1478-3 |
| 50 | Healthcare services research on pain in Germany. A survey | German | Article | 10.1007/s00482-015-0033-1 |
| 51 | Pain medicine from intercultural and gender-related perspectives | German | Article | 10.1007/s00482-015-0038-9 |
| 52 | Postoperative pain therapy in Germany. Status quo | German | Article | 10.1007/s00482-015-0039-8 |
| 53 | Survey of pain after ambulatory surgery | German | Article | 10.1007/s00482-015-0071-8 |
| 54 | QUIPSambulant. An instrument for quality assurance in acute pain therapy after outpatient operations | German | Article | 10.1007/s00482-015-1519-6 |
| 55 | Is supplemental ear acupuncture for surgical tooth removal with local anesthesia effective? A pilot study | German | Article | 10.1007/s00482-017-0212-3 |
| 58 | Ketamine in Pain Therapy | German | Article | 10.1007/s00482-019-0364-4 |
| 59 | Treatment expectations for postoperative pain | German | Article | 10.1007/s00482-021-00575-0 |
| 60 | Physical complaints and psychosocial stress in patients with endometrial cancer | German | Article | 10.1007/s00761-016-0154-x |
| 61 | The fast-track concept in treatment of critical ischemia of the extremities | German | Article | 10.1007/s00772-012-1100-x |
| 62 | Erythromelalgia after meniscus operation | German | Article | 10.1007/s00772-016-0230-y |
| 63 | Perioperative acute pain therapy. Does it have to hurt so much? | German | Article | 10.1007/s00772-016-0242-7 |
| 64 | Total elbow arthroplasty. Indications, procedures and results | German | Article | 10.1007/s10039-017-0283-5 |
| 65 | Sports Device Assisted Cardiac Rehabilitation Exercise Training After Coronary Artery Bypass Surgery | German | Article | 10.1055/a-1153-9125 |
| 66 | Perioperative Pain Management Patient Information, Informed Consent and Discharge Management | German | Article | 10.1055/a-1188-2591 |
| 67 | Temporarily Transfixation of the distal interphalangeal Joint in Mallet fingers | German | Article | 10.1055/a-1551-3481 |
| 68 | Hybrid NOS Appendectomy (NA): Clinical Assessment of a New Surgical Technique | German | Article | 10.1055/s-0032-1327892 |
| 69 | Transfer Managment of Postoperative Acute Pain Therapy to Outpatient Aftercare | German | Article | 10.1055/s-0033-1349840 |
| 70 | Intussusception after Billroth II operation fifty years ago - an unusual cause for ileus | German | Article | 10.1055/s-0033-1359883 |
| 71 | Avulsion of the Proximal Hamstring Origin - Report of 6 Cases | German | Article | 10.1055/s-0033-1360274 |
| 72 | Tonsillitis and Sore Throat in Childhood | German | Article | 10.1055/s-0033-1363210 |
| 73 | Pain Management in Video-Assisted Thoracic Surgery (VATS) | German | Article | 10.1055/s-0034-1383030 |
| 74 | Acute Pain Therapy for Appendectomy | German | Article | 10.1055/s-0043-104599 |
| 75 | Angina Pectoris in a Young Woman with Lupus Erythematosus | German | Article | 10.1055/s-0043-112074 |
| 76 | The Indication for Acute or Elective Surgery | German | Article | 10.1055/s-0043-121461 |
| 77 | Pecs II-block in breast cancer surgery: a pilot study on clinical routine procedures | German | Article | 10.19224/ai2019.301 |
| 78 | Quo vadis OPS 8-919? An analysis of coding and its relevance in clinical routine | German | Article | 10.19224/ai2021.146 |
| 79 | Perioperative analgesia with nonopioid analgesics | German | Article | 10.19224/ai2021.345 |
| 80 | Day surgery for inguinal hernia | German | Review | 10.1024/0040-5930/a001132 |
| 81 | Sequelae of hernia repair: What are the key issues? | German | Review | 10.1024/0040-5930/a001133 |
| 82 | Opioid crisis: What now? A pain specialist's point of view | German | Review | 10.1024/0040-5930/a001143 |
| 83 | Optimal Postoperative Pain Management After Tonsillectomy: An Unsolved Problem | German | Review | 10.1055/s-0041-107985 |
| 84 | Pain intensity and management after surgical procedures | German | Review | 10.19224/a12017.680 |
| 85 | Non-opioid analgesics in perioperative pain therapy | German | Review | 10.19224/ai2019.065 |
| 86 | Twisted pedunculated subserosal uterine leiomyoma: a case report | Greek | Article | 10.11212/exronika/2018.2.11 |
| 87 | Rare case of myocardial infarct in a young adult. Case report | Hungarian | Article | 10.1556/650.2015.30161 |
| 88 | The role of acute pain service in postoperative pain relief | Hungarian | Article | 10.1556/650.2020.31680 |
| 89 | Brain activation in non-human primate pain model using functional MRI | Japanese | Article | 10.11154/pain.35.45 |
| 90 | Factors Related to Persistent Postoperative Pain after Cardiac Surgery: A Systematic Review and Meta-Analysis | Korean | Review | 10.4040/jkan.2020.50.2.159 |
| 91 | Ileal strangulation around mesovarium in a newborn foal | Polish | Article | 10.21521/mw.6288 |
| 92 | Infrared image monitoring of local anesthetic poisoning in rats | Portuguese | Article | 10.1016/j.bjan.2016.02.004 |
| 93 | Analgesia Nociception Index: assessment of acute postoperative pain | Portuguese | Article | 10.1016/j.bjan.2019.01.003 |
| 94 | Articular and cutaneous nerve block at elbow: two cases report | Portuguese | Article | 10.1016/j.bjan.2020.03.014 |
| 95 | The effect of emotional stressors on postoperative skin conductance indices: a prospective cohort pilot study | Portuguese | Article | 10.1016/j.bjan.2020.04.009 |
| 96 | Cervical Spinal Cord Surgical Stabilization in a Sheep | Portuguese | Article | 10.22456/1679-9216.108515 |
| 97 | Vertebral Fractures and Luxation in Dogs | Portuguese | Article | 10.22456/1679-9216.95140 |
| 98 | Risk factors for pre, intra, and postoperative hospital mortality in patients undergoing aortic surgery | Portuguese | Article | 10.5935/1678-9741.20130004 |
| 99 | Calcitonin as an analgesic agent: review of mechanisms of action and clinical applications | Portuguese | Review | 10.1016/j.bjan.2019.08.004 |
| 100 | Transdermal buprenorphine for acute postoperative pain: a systematic review | Portuguese | Review | 10.1016/j.bjan.2020.04.004 |
| 101 | Modification to stapled mucosectomy technique with PPH. Experience of a surgical group | Spanish | Article | 10.1016/j.circir.2015.04.007 |
| 102 | Laparoscopic gastric sleeve in gastric volvulus secondary to diaphragmatic eventration in an adult patient | Spanish | Article | 10.1016/j.circir.2015.06.010 |
| 103 | Amyand's hernia and complicated appendicitis; case presentation and surgical treatment choice | Spanish | Article | 10.1016/j.circir.2015.06.012 |
| 104 | Results of the first 100 single port laparoscopic cholecystectomies in a secondary care hospital | Spanish | Article | 10.1016/j.ciresp.2013.03.014 |
| 105 | Quilting sutures compared with sponge packing in septolplasty and turbinoplasty | Spanish | Article | 10.1016/j.otorri.2020.09.001 |
| 106 | Postoperative delirium in patients with history of alcohol abuse | Spanish | Article | 10.1016/j.redar.2016.07.009 |
| 107 | Regional analgesia in postsurgical critically ill patients | Spanish | Article | 10.1016/j.redar.2016.09.012 |
| 108 | Pharmacological advances in the multimodal management of perioperative analgesia | Spanish | Article | 10.1016/j.redar.2017.03.006 |
| 109 | Erector spinae plane block: A cadaver study to determine its mechanism of action | Spanish | Article | 10.1016/j.redar.2018.07.004 |
| 110 | BRILMA block for costal cartilage excision: Case report | Spanish | Article | 10.1016/j.redar.2020.01.009 |
| 111 | Postoperative lactate elevation as a marker of underlying acute mesenteric ischemia. Description of two cases | Spanish | Article | 10.1016/j.redar.2020.08.010 |
| 112 | Case report. Pelvic inflammatory disease as a complication of acute appendicitis | Spanish | Article | 10.17533/udea.iatreia.84 |
| 113 | Role of central sensitization in the chronification of postoperative pain | Spanish | Article | 10.20960/rhh.125 |
| 114 | Bowel obstruction due to video capsule endoscopy in a patient with Crohn's disease | Spanish | Article | 10.24875/CIRU.19000710 |
| 115 | Intraperitoneal prolapse of the amniotic sac. A case report | Spanish | Article | 10.31403/rpgo.v64i2087 |
| 116 | Anesthesia, analgesia, rheumatology and relief of acute and chronic pain | Spanish | Article | 10.5281/zenodo.2554484 |
| 117 | Groove pancreatitis vs. pancreatic adenocarcinoma: A review of 8 cases | Spanish | Review | 10.1016/j.ciresp.2016.02.011 |
| 118 | A Comparison of Pregabalin and Ketamine in Acute Pain Management After Laparoscopic Cholecystectomy | Turkish | Article | 10.14235/bs.2017.1314 |
| 119 | Evaluation of patients with spontaneous pneumomediastinum diagnosis in the emergency department | Turkish | Article | 10.17826/cumj.508742 |
| 120 | Comparison of four different pain scales in the evaluation of postoperative acute pain intensity | Turkish | Article | 10.17826/cumj.741465 |
| 121 | Long-term Results of in Situ Pinning Treatment of Femoral Head Slippage Patients | Turkish | Article | 10.5152/imj.2018.09815 |
| 122 | Multimodal analgesia for pediatric patients who underwent open or laparoscopic appendectomy | Turkish | Article | 10.5222/buchd.2018.095 |
| 123 | Intestinal perforation due to multiple magnet ingestion: a case report | Turkish | Article | 10.5505/tjtes.2012.22457 |
|  |  |  |  |  |

**Supplementary table 3 Details of literature excluded due to type of literature**

| **Rank** | | **Article Title** | **Language** | **Document Type** | **DOI** |
| --- | --- | --- | --- | --- | --- |
| 1 | A review of postoperative pain assessment records of nurses (vol 38, pg 1, 2017) | | English | Correction | 10.1016/j.apnr.2018.01.001 |
| 2 | Intranasal ketorolac for acute postoperative pain (vol 26, pg 1915, 2010) | | English | Correction | 10.1185/03007995.2012.689203 |
| 3 | Perioperative ketamine for acute postoperative pain (Withdrawn paper. 2015, art no. CD004603) | | English | Correction | 10.1002/14651858.CD004603.pub3 |
| 4 | Single dose dipyrone for acute postoperative pain (Withdrawn Paper. 2013, art. no CD003227) | | English | Correction | 10.1002/14651858.CD003227.pub3 |
| 5 | A Case of Duodenal Obstruction and Pancreatitis Due to Intragastric Balloon | | English | Editorial Material | 10.5152/balkanmedj.2015.15312 |
| 6 | ACOEM Practice Guidelines: Opioids for Treatment of Acute, Subacute, Chronic, and Postoperative Pain | | English | Editorial Material | 10.1097/JOM.0000000000000352 |
| 7 | Acute and chronic neuropathic pain after surgery Still a lot to learn | | English | Editorial Material | 10.1097/EJA.0000000000000682 |
| 8 | Acute Aortic Dissection Extending Into the Lung | | English | Editorial Material | 10.1016/j.athoracsur.2014.08.060 |
| 9 | Acute appendicitis: What is the gold standard of treatment? | | English | Editorial Material | 10.3748/wjg.v19.i47.8799 |
| 10 | Acute Management of Infected Chronic Thromboembolic Disease | | English | Editorial Material | 10.1016/j.athoracsur.2018.04.006 |
| 11 | Acute neuropathic pain and the transition to chronic postsurgical pain | | English | Editorial Material | 10.2217/pmt-2018-0026 |
| 12 | Acute Pain Management in Patients Treated With Buprenorphine: A Teachable Moment | | English | Editorial Material | 10.1001/jamainternmed.2019.3103 |
| 13 | Acute Postoperative Pain Control | | English | Editorial Material | 10.1155/2017/7831014 |
| 14 | Adjuvants to local anesthetics: Current understanding and future trends | | English | Editorial Material | 10.12998/wjcc.v5.i8.307 |
| 15 | Advanced malignant mesothelioma mimicking acute contained thoracic aortic rupture | | English | Editorial Material | 10.1093/icvts/ivt465 |
| 16 | An up-to-date overview of sublingual sufentanil for the treatment of moderate to severe pain | | English | Editorial Material | 10.1080/14656566.2020.1766025 |
| 17 | Anorectal emergencies | | English | Editorial Material | 10.3748/wjg.v22.i26.5867 |
| 18 | Buprenorphine for the management of acute pain | | English | Editorial Material | 10.1177/0310057X1704500202 |
| 19 | Cannabis, anesthesia and acute postoperative pain: known and unknown | | English | Editorial Material | 10.2217/pmt-2020-0096 |
| 20 | Cochrane in CORR (R): Perioperative Intravenous Ketamine for Acute Postoperative Pain in Adults | | English | Editorial Material | 10.1097/CORR.0000000000000981 |
| 21 | Continuous wound infiltration of local anaesthetics for acute postoperative pain - A revisit | | English | Editorial Material | 10.4103/ija.IJA_425_19 |
| 22 | Coronary artery aneurysm occurring very late after drug-eluting stent implantation | | English | Editorial Material | 10.1093/icvts/ivu286 |
| 23 | Dissecting Thoracic Aneurysm in Takayasu Arteritis With Concomitant Tuberculosis | | English | Editorial Material | 10.1016/j.athoracsur.2019.05.014 |
| 24 | Do we still need gabapentinoids in anaesthesia? | | English | Editorial Material | 10.1016/j.accpm.2021.100923 |
| 25 | Efficacy and safety of dual opioid therapy | | English | Editorial Material | 10.1517/14740338.2014.966077 |
| 26 | Fascial plane blocks: the next leap | | English | Editorial Material | 10.1136/rapm-2020-101992 |
| 27 | Guidelines for the use of buprenorphine for opioid use disorder in the perioperative setting | | English | Editorial Material | 10.1136/rapm-2021-103092 |
| 28 | Hot Off the Press: Subdissociative-dose Ketamine for Acute Pain in the Emergency Department | | English | Editorial Material | 10.1111/acem.12705 |
| 29 | Important considerations with respect to reducing the transition from acute to persistent postoperative pain | | English | Editorial Material | 10.1080/14656566.2021.1892073 |
| 30 | Incarcerated umbilical Littre's hernia at the trocar site of a previous laparoscopic surgical procedure | | English | Editorial Material | 10.1308/rcsann.2016.0133 |
| 31 | Intussusception After Roux-en-Y Gastric Bypass: Laparoscopic Management | | English | Editorial Material | 10.1007/s11695-018-3265-1 |
| 32 | Intussusception in the Setting of Tuberous Sclerosis Complex | | English | Editorial Material | 10.14309/crj.0000000000000244 |
| 33 | Ising Model of the Chronification of Acute Pain | | English | Editorial Material | 10.1111/pme.12932 |
| 34 | Laparoscopic appendectomy in a pediatric patient with type 1 Charcot-Marie-Tooth disease | | English | Editorial Material | 10.1016/j.jclinane.2015.07.021 |
| 35 | Low-Dose Ketamine for Acute Pain Management: A Timely Nudge Toward Multimodal Analgesia | | English | Editorial Material | 10.1097/AAP.0000000000000810 |
| 36 | Man with chest pain | | English | Editorial Material | 10.1002/emp2.12296 |
| 37 | Management of acute pain in the postoperative setting: the importance of quality indicators | | English | Editorial Material | 10.1080/03007995.2017.1391081 |
| 38 | Methadone and Ketamine: Boosting Benefits and Still More to Learn | | English | Editorial Material | 10.1097/ALN.0000000000003752 |
| 39 | Minimizing Excess Opioid Prescribing for Acute Postoperative Pain | | English | Editorial Material | 10.1001/jamaoto.2019.4209 |
| 40 | Nerve-sparing laparoscopic treatment of parametrial ectopic pregnancy | | English | Editorial Material | 10.1016/j.fertnstert.2021.05.106 |
| 41 | No disputes when there is a simple solution: monitoring in acute compartment syndrome | | English | Editorial Material | 10.1111/anae.15516 |
| 42 | Non-Invasive and Minimally Invasive Management of Low Back Disorders | | English | Editorial Material | 10.1097/JOM.0000000000001812 |
| 43 | Optimal Organization of Acute Pain Services: At the Confluence of Semantics, Logistics, and Economics | | English | Editorial Material | 10.1213/ANE.0000000000003367 |
| 44 | Pain after surgery-acute becomes chronic: might there be a silver ROCKet? | | English | Editorial Material | 10.1177/0310057X1704500404 |
| 45 | Pre-, intra-, and postoperative management of Robert's uterus | | English | Editorial Material | 10.1016/j.fertnstert.2018.05.033 |
| 46 | Prevention of Venous Thromboembolism in Gynecologic Surgery ACOG Practice Bulletin, Number 232 | | English | Editorial Material | 10.1097/AOG.0000000000004446 |
| 47 | Procedure-specific Pain Management The Road to Improve Postsurgical Pain Management? | | English | Editorial Material | 10.1097/ALN.0b013e31828866e1 |
| 48 | Should This Patient Receive Prophylactic Medication to Prevent Delirium? | | English | Editorial Material | 10.7326/M18-0388 |
| 49 | The Perioperative Surgical Home: A New Role for the Acute Pain Service | | English | Editorial Material | 10.1213/ANE.0000000000002165 |
| 50 | The problem of postoperative respiratory depression | | English | Editorial Material | 10.1111/jcpt.13382 |
| 51 | The time course of acute pain in hospitalized patients: exciting progress in data and methods | | English | Editorial Material | 10.1097/j.pain.0000000000000714 |
| 52 | Thrombosed aneurysm of saphenous vein coronary artery bypass grafting | | English | Editorial Material | 10.1590/1806-9282.63.06.488 |
| 53 | Transapical Endovascular Stenting of Penetrating Atherosclerotic Ulcer of Ascending Aorta | | English | Editorial Material | 10.1016/j.athoracsur.2013.03.106 |
| 54 | Undersized Stent Grafts for Acute Mesenteric Ischemia in Chronic Type B Dissection | | English | Editorial Material | 10.1016/j.athoracsur.2016.12.038 |
| 55 | Unusual cause of acute abdomen in a child - torsion of greater omentum: report of two cases | | English | Editorial Material | 10.1177/0036933015581129 |
| 56 | Using acute pain scales for cats | | English | Editorial Material | 10.1136/vr.j2065 |
| 57 | When acute pain becomes chronic | | English | Editorial Material | 10.1111/anae.13145 |
| 58 | Why is there no morphine concentration-response curve for acute pain? | | English | Editorial Material | 10.1111/pan.12361 |
| 59 | Acute and chronic postsurgical pain after living liver donation: Incidence and predictors | | English | Letter | 10.1002/lt.24125 |
| 60 | Acute and Chronic Postsurgical Pain After Living Liver Donation: Incidence and Predictors Reply | | English | Letter | 10.1002/lt.24177 |
| 61 | Acute Postoperative Pain Trajectory Groups: Comment | | English | Letter | 10.1097/ALN.0000000000003875 |
| 62 | Acute Postoperative Pain Trajectory Groups: Reply | | English | Letter | 10.1097/ALN.0000000000003874 |
| 63 | Analgesia/nociception index for the assessment of acute postoperative pain | | English | Letter | 10.1093/bja/aeu116 |
| 64 | Association of age with accuracy of surgical pleth index to predict major postoperative pain | | English | Letter | 10.1016/j.bja.2019.10.015 |
| 65 | Blockade of femoral and obturator nerves by a pubic muscle plane block | | English | Letter | 10.1016/j.jclinane.2019.05.019 |
| 66 | Can intraoperative Surgical Pleth Index values be predictive of acute postoperative pain? | | English | Letter | 10.1016/j.accpm.2018.05.004 |
| 67 | Cannabinoids and acute/postoperative pain management | | English | Letter | 10.1097/j.pain.0000000000002294 |
| 68 | Cannabinoids and acute/postoperative pain management Reply | | English | Letter | 10.1097/j.pain.0000000000002295 |
| 69 | Confounding Factors in Predicting Acute Postsurgical Pain | | English | Letter | 10.1111/papr.12757 |
| 70 | Erector spinae plane block for postoperative analgesia in kidney transplantation: A report of 3 cases | | English | Letter | 10.1016/j.jclinane.2019.109683 |
| 71 | Erector spinae plane block: systemic local anesthetic by proxy? | | English | Letter | 10.1136/rapm-2020-102443 |
| 72 | Minimal clinically important difference: a context-specific metric | | English | Letter | 10.1136/rapm-2020-102330 |
| 73 | Pericapsular nerve group block for postoperative shoulder pain: A cadaveric radiological evaluation | | English | Letter | 10.1016/j.jclinane.2020.110058 |
| 74 | Possible confounders in study of oral versus i.v. acetaminophen for postoperative pain control | | English | Letter | 10.2146/ajhp180273 |
| 75 | Preemptive analgesia for acute postoperative pain management in children | | English | Letter | 10.1111/pan.12942 |
| 76 | Reconstructive surgery and persistent postsurgical pain after mastectomy | | English | Letter | 10.1016/j.breast.2018.01.009 |
| 77 | ACUTE PAIN ASSESSMENT IN SEDATED POSTOPERATIVE PATIENTS | | English | Meeting Abstract |  |
| 78 | An evaluation of acute postoperative pain management in patients from different ethnicities | | English | Meeting Abstract |  |
| 79 | Animal Models of Acute, Inflammatory, and Postoperative Pain | | English | Meeting Abstract | 10.1016/j.jpain.2015.01.189 |
| 80 | Audit of efficacy of postoperative acute pain management for total knee replacements | | English | Meeting Abstract |  |
| 81 | Comparative Effectiveness of CL-108 and Ibuprofen for Acute Postoperative Pain | | English | Meeting Abstract |  |
| 82 | Does ethnicity affect acute postoperative pain and nurses attitudes to treating it? | | English | Meeting Abstract |  |
| 83 | POSTOPERATIVE PAIN EXPERIENCE: RESULTS FROM A HOSPITAL ACUTE PAIN SERVICE | | English | Meeting Abstract |  |
| 84 | Safer and More Effective Treatment of Acute Postoperative Pain in Living Liver Donors | | English | Meeting Abstract |  |
| 85 | Safety of epidural analgesia for acute postoperative pain in Ealing Hospital 2015-2016 | | English | Meeting Abstract |  |
| 86 | Acute pain and migraine: the evidence | | English | Proceedings Paper |  |
| 87 | Acute Pain In Craniofacial Trauma | | English | Proceedings Paper |  |
| 88 | Acute Pain Management After Direct Laryngoscopy in Early Laryngeal Cancers | | English | Proceedings Paper |  |
| 89 | Advances in Monitoring Postoperative Recovery | | English | Proceedings Paper |  |
| 90 | Current Approach in Treating the Postoperative Pain in Thoracic Surgery | | English | Proceedings Paper |  |
| 91 | Deep Neural Network Architectures for Forecasting Analgesic Response | | English | Proceedings Paper |  |
| 92 | Hand Pain in Rehabilitation Following Upper Limb Surgery | | English | Proceedings Paper |  |
| 93 | Management of Postoperative Pain in Newborns | | English | Proceedings Paper |  |
| 94 | Modeling and Analysis of Monitored vs. Self-reported Postsurgical Acute Pain in a Clinical Trial | | English | Proceedings Paper | 10.1016/j.ifacol.2021.10.233 |
| 95 | Pain assessment in surgical patients with impaired cognition | | English | Proceedings Paper | 10.15405/epsbs.2016.07.02.13 |
| 96 | Pain in Tonsillectomy | | English | Proceedings Paper |  |
| 97 | Chronic Pain Syndromes, Mechanisms, and Current Treatments | | English | Book Chapter | 10.1016/bs.pmbts.2015.01.004 |
| 98 | Opioid Receptors | | English | Book Chapter | 10.1146/annurev-med-062613-093100 |
